# Multiple cerebral infarction revealing Takayasu's disease: a case report in a 32‐year‐old man from Cameroon, sub‐Saharan Africa

**DOI:** 10.1002/ccr3.1380

**Published:** 2018-02-07

**Authors:** Félicité Kamdem, Caroline Kenmegne, Ba Hamadou, Yacouba Mapoure, Fernando K. Lekpa, Sidicki Mouliom, Ahmadou Musa Jingi, Henry Luma, Marie Solange Doualla

**Affiliations:** ^1^ Internal Medicine Service Douala General Hospital Douala Cameroon; ^2^ Faculty of Medicine and Pharmaceutical Sciences University of Douala Douala Cameroon; ^3^ Faculty of Medicine and Biomedical Sciences University of Yaounde 1 Yaounde Cameroon

**Keywords:** Africa, Cameroon, cerebral infarction, stroke, Takayasu's disease

## Abstract

This case suggests that young patients with few vascular risk factors, and who present with acute stroke syndrome involving more than one vascular territory should be screened for an inflammatory or infectious cause.

## Introduction

Takayasu's disease is a chronic inflammation of the large vessels that principally affects the aorta and its main branches. It is a rare disease that is seen in 1.2 cases per million inhabitants per year in Europe and 2.6 cases per million inhabitants per year in North America 1, 2. The highest prevalence is seen in South East Asia, India, and South America. It is rare in blacks 3. Women are more affected than men – 90% of cases 4. Thickening of the vascular wall is an early sign that characterizes the disease, which progressively leads to stenosis, thrombosis, and at times the formation of aneurysms. The evolution is in two phases – systemic and occlusive. The systemic inflammatory phase often goes un‐noticed, and it is only evoked retrospectively during interview. We report the case of Takayasu's disease in a 32‐year‐old man with no vascular risk factors from Cameroon – sub‐Saharan Africa, who presented with an acute stroke syndrome. CT scan of the brain showed multiple cerebral infarctions. The aim of this report is to guide the diagnostic approach and management of acute stroke syndromes in young Africans. We report this case in accordance with the CARE guideline.

## Case Presentation

**Figure 1:**
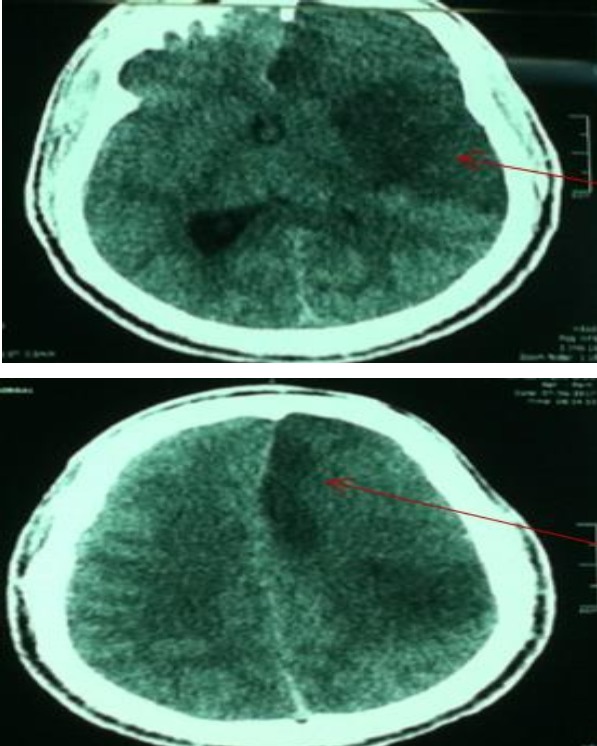
CT scan of the brain showing multiple cerebral infarctions.

**Figure 2:**
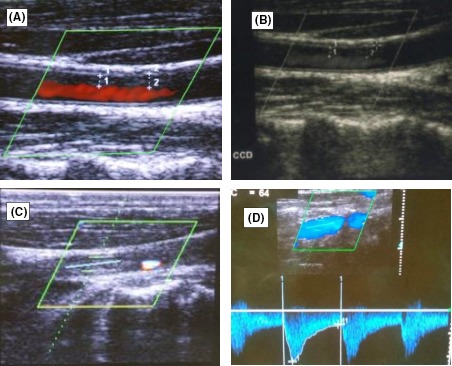
Doppler ultrasound of the neck vessels showing inflammatory wall thickening (A and B), thrombotic occlusion of the left common carotid artery (C), and flow reversal of the vertebral artery (D).

**Figure 3:**
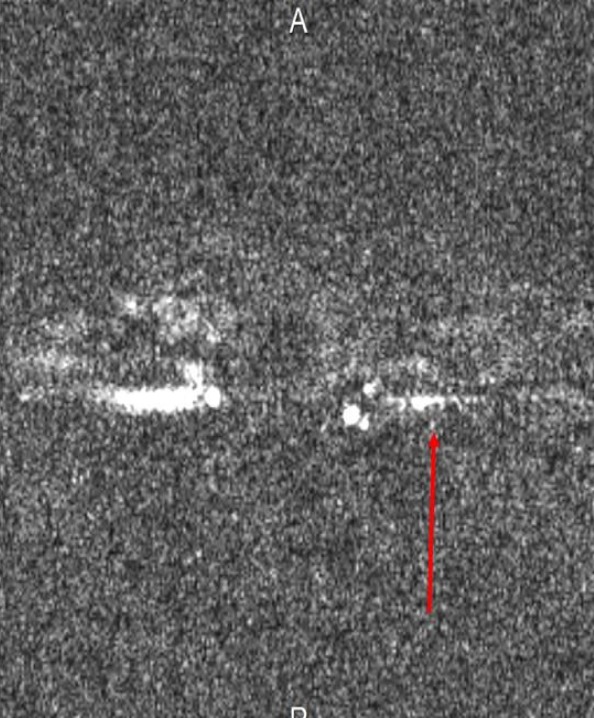
Angio‐CT of the neck vessels showing left subclavian artery stenosis.

**Figure 4:**
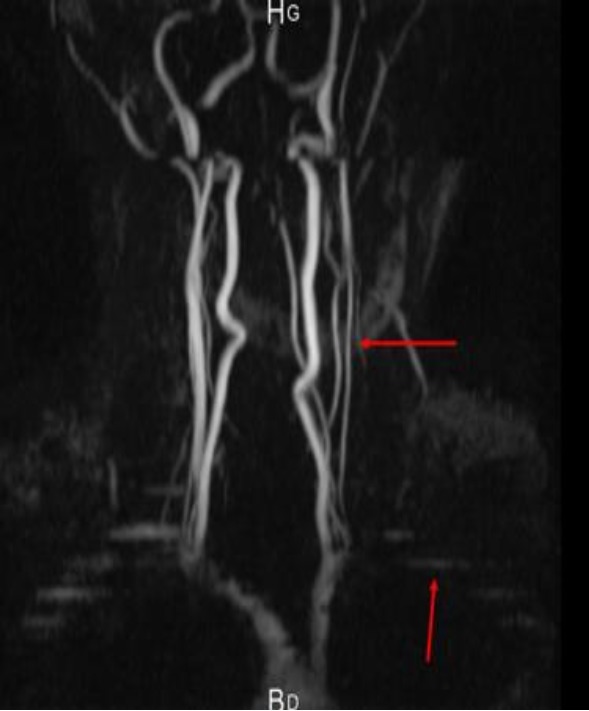
Angio‐MRI of the neck and head vessels showing left vertebral artery constriction and left subclavian artery stenosis.

**Figure 5:**
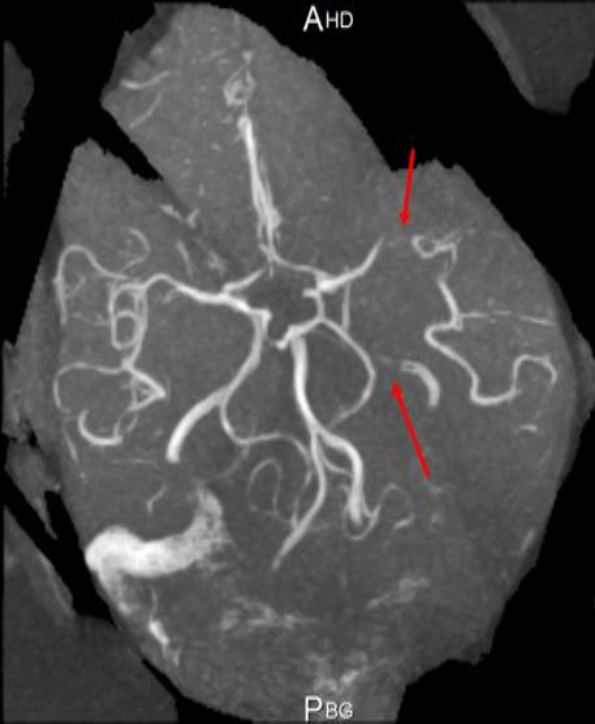
Angio‐MRI of the brain showing left carotid artery constriction.

A 32‐year‐old man with no vascular risk factor was hospitalized in June 2017 in the Internal Medicine unit of the Douala General Hospital (DGH) – Cameroon, for the management of sudden onset right hemiplegia, associated with aphasia. This has been evolving since 1 week before his admission. On clinical examination, he was conscious and afebrile and had asymmetry of the brachial blood pressure measurements: 142/71 mmHg on the left arm and 125/80 mmHg on the right arm. The left radial and femoral pulses were reduced in volume. He had vascular claudication of the left arm and right hemiplegia. He had bilateral carotid artery and abdominal bruits on auscultation. Cardiac ultrasound was unremarkable. Blood analysis showed inflammation – ESR: 87 mm in the first hour and CRP: 12 mg/L. Full blood count, liver enzymes, lipid profile, renal function, and fasting blood glucose were normal. HIV, hepatitis B surface antigene, hepatitis C antibodies, and Syphilis serology were negative. The search for tuberculosis was negative on GeneXpert. Cerebrospinal fluid analysis showed an inflammatory fluid – Proteinorrachia=CSF protein 1.06 g/L, Glucorrachia=CSF glucose 0.66 g/L, with no cells. India ink staining was negative for cryptococcoses. Cerebral CT scan (Fig. 1) showed multiple cerebral infarcts in the region of the left anterior cerebral and superficial middle cerebral arteries. Doppler ultrasound of the neck vessels (Fig. 2) showed thrombosis of the left common carotid artery, about 12 mm to the bifurcation. There was no hemodynamic stenosis. There was inversion of the vertebral artery flow, with vertebral steal phenomenon. There was acceleration of flux at the level of the subclavian artery. There was a hypoechoic plaque of 3 mm at the level of the right common carotid artery, with no hemodynamic stenosis. MRI of the neck vessels showed inflammatory stenosis of the left subclavian and left vertebral arteries (Figs. 3 and 4). There was no evidence of dissection of the neck vessels. Angio‐MRI of the brain (Fig. 5) showed constricted arteries. Cardiac ultrasound and electrocardiography were normal. The diagnosis of Takayasu's arteritis was made based on the American College of Rheumatology criteria (Table 1). IV bolus of methylprednisone (1 mg/kg/day) was administered, associated with aspirin. The patient underwent motor and speech re‐education. On discharge, after 15 days of hospitalization, he remained conscious and hemiplegic. He was lost to follow‐up.

**Table 1:** American College of Rheumatology (ACR) diagnostic criteria (patient's clinical findings)

Age at onset ≤ 40 ans. Vascular claudication of the left arm. Decreased brachial artery pulse. Systolic blood pressure difference > 10 mmHg between arms Presence of vascular bruits over subclavian artery and abdomen. Arteriographic abnormality: stenosis or occlusion of the aorta or its main branches not due to atherosclerosis or fibromuscular dysplasia.

NB: The presence of ≥3 criteria has a sensitivity of 90.5% and specificity of 97.8%.

## Discussion

We report the case of Takayasu's arteritis in a 32‐year‐old man with no vascular risk factor. The initial presentation was an acute stroke syndrome. CT scan of the brain showed more than two vascular territory involvements. The importance of this case is to guide our attitude toward the management of young patients with an acute stroke syndrome in a low‐income setting. Inflammatory and infectious causes of acute stroke should be considered as these determine treatment and outcome.

Takayasu's disease is a chronic inflammation of the big vessels – the aorta and its main branches, that affects young women in 90% of cases. Muscle involvement is rare, with few cases reported in the literature 5. The preocclusive phase is characterized by systemic symptoms and inflammation. The occlusive phase is characterized by the occurrence of ischemic manifestations. Neurologic manifestations of the disease are polymorphic. They are due to transitory or permanent central nervous system ischemia as a result of neck vessel involvement 6, 7. Arnaud et al. showed that the occurrence of neurological involvement was 10 times higher in non‐Caucasians than in Caucasians 8. Peripheral manifestations are essentially due to stenosis of the arterial tree. Reduced or absence of peripheral pulses, as well as the gradual onset of vascular claudication, is the most frequently reported early sign of the disease 5, 6, 9, 10. The presence of vascular bruits in the neck vessels, the subclavian region, and the abdomen is frequently reported 5, 6, 9, 10. Hypertension is frequent, at times revealing the disease. This is, however, underestimated due to vascular stenosis 6, 11. When hypertension is present in the setting of acute stroke, it could lead to confusion as it is a risk factor for stroke. In such scenarios, it could be a physiologic response due to acute stroke, or it could be secondary to aortic or renal artery stenosis 1. The asymmetry of blood pressure measurements or pulse deficit could help in making the diagnosis. On Doppler ultrasound, a homogeneous and circumferential thickening of the vessel walls of the neck, subclavian, and abdominal arteries not consistent with atherosclerosis is in favor of Takayasu arteritis 12, 13, 14. At least three of the five criteria of the American College of Rheumatology (ACR) are needed to make the diagnosis, with a sensitivity of 90% and a specificity of 98% (Table 1). Corticosteroids are classically the first line of treatment with good response 10, 15, 16, 17. However, up to one‐fourth of patients with dual immunosuppressive medicines failed to have disease remission 1. Our case had multiple cerebral infarctions which did not show improvement on discharge at day 15 of hospitalization. This was probably due to the large and multiple cerebral infarcts. The patient was lost to follow‐up after discharge. We could not assess the neurological and vascular recovery after discharge.

As limitations, we did not test for autoantibodies nor perform arterial biopsy. Autoantibody testing is not readily available in our setting. Arterial biopsy is invasive. However, the patient fulfilled all the ACR diagnostic criteria of Takayasu's arteritis.

## Conclusion

This case suggests that young patients with few vascular risk factors, and who present with acute stroke syndrome involving more than one vascular territory, should be screened for an inflammatory or infectious cause. Takayasu's disease is arteritis of the large vessels – aorta and its main branches. The clinical presentation is polymorphic. The diagnosis is based on clinical suspicion and vascular imaging according to the American College of Rheumatology criteria. Corticosteroids are the first line of treatment.

## Ethical Statement

The patient's closest relative gave informed consent for this case to be published. The ethical committee of the DGH approved the publication of this case. The patient is still receiving regular care at the Douala General Hospital.

## Authorship

All the authors were involved in patient care. FK and CK: drafted the manuscript. All the authors critically reviewed and approved the final draft for publication.

## Conflict of Interest

None declared.
